# Relationship between Individual Social Capital and Functional Ability among Older People in Anhui Province, China

**DOI:** 10.3390/ijerph17082775

**Published:** 2020-04-17

**Authors:** Zhongliang Bai, Zijing Wang, Tiantai Shao, Xia Qin, Zhi Hu

**Affiliations:** 1 Department of Epidemiology and Biostatistics, School of Public Health, Anhui Medical University, Hefei 230032, China; 2 Department of Health Services Management, School of Health Services Management, Anhui Medical University, Hefei 230032, China

**Keywords:** social capital, older people, health, disability

## Abstract

This study aimed to explore the relationship between individual social capital and functional ability, with a focus on whether there is an interactive relationship that exists among social capital related to functional ability among older people in Anhui province, China. We conducted a cross-sectional study with a multi-stage stratified cluster random sampling method from July to September 2017. Data were collected through questionnaire including demographic characteristics, individual social capital status, and functional capability status. Binary logistic regression analysis model and classification and regression tree model (CART) were utilized. Overall, this study included 1810 elderly people, 43% of whom had functional disability. After the adjustment, subjects with lower social participation (AOR = 1.60; 95% CI: 1.26–2.03) and lower social connection (AOR = 1.74; 95% CI: 1.34–2.25) had an increased risk of functional disability. However, social support (AOR = 0.73; 95% CI: 0.57–0.94) was inversely related to functional ability. We also observed interactive relationship of social capital associated with functional ability, which indicated that special attention and efforts should be paid to older adults with less educational attainment, with multimorbidity, with advanced age, and with lower level of social participation, cohesion for the purpose of maintaining sound functional ability. Our findings may be of salient relevance for devising more targeted and effective interventions to prevent the onset of functional limitations among community-dwelling older adults.

## 1. Introduction

With a rapidly aging population, more and more countries face huge challenges posed by this demographic shift to the family, social, and health systems [1]. By the end of 2018, China had a population of 249 million aged 60 or above, accounting for 17.9% of the national population [2]. Previous studies have shown that elderly functional disability, characterized by activities of daily living (ADL) and instrument activities of daily living (IADL) limitations, is a major public health issue faced by many countries, including China [3]. It has been found that limitations in ADL or IADL function not only deteriorate the ability to live independently, but also reduce the quality of life of elderly individuals [3]. However, it remains a challenge thing about how to maintain the elderly functional capability [4].

The World Health Organization has reported that about 15.0% of the world’s population suffers from functional disability to a different extent [5]. Several studies have indicated that the prevalence of functional limitations or disability is high and varies across countries. For example, the prevalence of functional limitations is about 22% in a study aimed at exploring the severity and factors among Netherlandish older adults with functional limitations [6]. A study of two birth cohorts of older adults (aged ˃ 75 years) in Sweden reported that the prevalence of limitations in ADL or IADL ranged from 13.0% to 37.5% [7]. In Asia, results derived from Global AGEing and Adult Health (SAGE) Wave 1 (2007–2010) found that the prevalence of functional disability among older people ranged from 16.2% in China to 55.7% in India [8].

Greater attention has been paid to exploring the correlates concerning the health and well-being of older adults [9]. Previous studies from China and abroad have both found that advanced age [10,11], frailty [12,13], depression [14] underweight or obesity, physical inactivity, a poor self-rated health, less engagement in level social activities, and pain [15] were most commonly associated with the incidence of ADL or IADL disability. Furthermore, many studies have identified that comorbidity accelerated the decline of functional ability of the elderly [3,11]. For instance, Brian and his colleagues [16] found that older Puerto Rican adults with comorbid diabetes and depressive symptoms were prone to developing ADL disability and mortality. Su et al. [3] found that among Chinese community-dwelling elderly people, multimorbidity (comorbid with chronic diseases) was significantly associated with ADL and IADL impairment; similar findings were also observed in a study conducted in Netherlands [17].

Studies have recently found that social capital, which reflects how individuals or groups can get resources by their inter-social networks and support, has substantial influence on elderly health [18]. The role of social capital in living arrangements [19], mental health [20], and life satisfaction was observed among elderly people from previous studies in China [21]. Moreover, the association between social capital and elderly functional ability was examined in multiple studies. For example, Gontijo et al. [22] found that poor social capital was associated with functional limitations in older Brazilian people. A prospective cohort study in Japan suggested that community dwellers with a higher level of social participation have less chance to be ADL disability [23]. Tomioka et al. [24] found that the more social participation (i.e., volunteer groups, hobby groups) involved, the healthier functional ability older adults could have. Similarly, Hao et al. [25] found that elderly with disability had more communication with others, were less likely to have ADL limitations among older Chinese adults.

Researchers have claimed that apart from health and medical conditions, the interactions between different correlates (i.e., health problems, individual factors, and engagement in activities) also contribute to the development of functional limitations among older adults [26]. Despite the fact that studies investigating multiple factors (including demographic and social capital) individually related to functional ability are of relevance, a comprehensive appreciation of the correlates associated with the performance of daily activities of life is of great importance to devise specific and appropriate strategies aimed to reduce the presence of functional decline in older adults. However, to our knowledge, such interactions are less explored and understood so far. In this scenario, this study aims to explore the relationship between individual social capital and functional capability, with a particular focus on whether there is an interactive relationship that exists among social capital dimensions related to functional ability among community-dwelling older people in Anhui province, China.

## 2. Materials and Methods

### 2.1. Study Design and Data Collection

This cross-sectional survey was part of the large-scale epidemiological study of social capital and healthy aging among older people in the Anhui province of China, which was conducted during July and September 2017. Ethical approval for this study was obtained from the Biomedical Ethics Committee in Anhui Medical University (No. 20150297).

A multi-stage stratified cluster random sampling method was applied to enroll participants for the purpose of having a good representative sample. First, we selected three prefecture-level cities from the sixteen prefecture-level cities in Anhui province: Fuyang (north); Xuancheng (south); Hefei (central, the capital of Anhui). Second, in each prefecture-level city, one county and one district were selected randomly. A total of six counties and districts were finally selected in this study. Next, in each selected county and district, one street community and one township were randomly selected, and a total of 12 street communities and townships were chosen. At the last stage, in each selected street community and township, two communities and two villages were selected randomly, and 24 sampling areas were confirmed.

According to the local existing household registration system data, individuals aged 60 and up were ascertained. With the assistance of local community workers, each participant was personally visited at their home and interviewed by skilled and trained graduate students from the Anhui Medical University face to face using a structured questionnaire. A verbal understanding of the purposes and procedures of the study and consent forms were needed before the interview. Individuals with whom the proper verbal communication was not possible, owing to reasons, for instance, severe deafness and poor communication skills, were excluded. In the present study, a total of 1935 older adults agreed to participate in the interview, among which 1810 who completed the whole survey procedure were eligible for analysis. In total, the response rate was 93.5% (1810 of 1935).

### 2.2. Measures

#### 2.2.1. Measurement of Functional Disability

In this study, a composite ADL/IADL scale, which was adapted from the 1969 study of American scholars Lawton and Brody [27], was used to assess the functional capability of the elderly sample. This scale contained 14 items that measure Activities of Daily Living (ADL) and Instrumental Activities of Daily Living (IADL). The eight items of ADL include eating, bathing, grooming, dressing, toileting, indoor activities, walking, and ascending and descending stairs. The six items of IADL encompassed using phone calls, shopping, doing laundry, using vehicles, taking pills, and saving or withdrawing money. Each item had three options, with scores ranging from 1 to 3 and respondents were asked to rate their agreement (1 = “independently”, 2 = “partially limited”, 3 = “dependently”). The total score was 14–42 and respondents were classified as having functional disability if he or she reported that they “partially limited or dependently” to any items. Functional ability was recorded dichotomously (limited versus robust). The Cronbach’s α of the questionnaire was 0.925. The results of this scale in China have been proven reliable and robust [9].

#### 2.2.2. Measurement of Social Capital

Based on the World Bank’s Social Capital Assessment Tool and previous works, social capital (including six dimensions, such as social participation, social support, social connection, trust, cohesion, and reciprocity) was included in the present study. We selected 22 commonly used and easily understood items to measure social capital and adapted them to the Chinese context [28,29].

In the present study, the five-point scale of Likert was adopted in the social capital questionnaire, and respondents were asked to rate their agreement (1 = “never”, 2 = “seldom”, 3 = “= “usually”, 4 = “often”, and 5 = “more often”). For each domain of social capital, answers to varied items were summarized to get an overall score, and a higher score indicates a better social capital status. In data analysis, we dichotomized the scores of each dimension social capital into two categories by taking the median value as the cut-off [30,31], including social participation(high (≥6) and low (<6)), social support(high (≥13) and low (<13)), social connection (high (≥12) and low (<12)), trust (high (≥13) and low (<13)), cohesion(high (≥20) and low (<20)), and reciprocity(high (≥11) and low (<11)). The Cronbach’s *α* of the questionnaire was 0.919.

#### 2.2.3. Measurement of other Variables

Information on the demographic and health-related variables was collected. These variables included name, age (60–64, 65–69, 70–74,75–79, ≥80 years), gender (male, female), body mass index (BMI, kg/m^2^): <18.5 = underweight, 18.5–22.9 = normal weight, 23.0–27.4 = overweight, and ≥27.5 = obese, residence (urban, rural), living status (living alone, living with others), marital status (married /cohabited, and single, including never married, divorced, and widowed), education (primary school and below, junior high school, high school and above). Based on the physical health section of the Older Americans Resources and Services (ORAS) [32,33], multimorbidity status (yes, no) of the participants was obtained. We asked participants whether they had received a doctor diagnosis of high blood pressure, diabetes, heart disease (coronary or valve disease), hyperlipidemia, angina, chronic bronchitis (COPD/emphysema), cerebral infarction (stroke), coronary heart disease, cataract, arthritis, dementia, cancer, depression, liver or kidney-related diseases. Participants were categorized into having multimorbidity group if they have at least two kinds of above-mentioned diseases. Meanwhile, information on smoking and drinking status were also collected.

### 2.3. Statistical Analysis

First, a Chi-squared test was employed to examine the difference between different functional capability groups (limited versus robust). The demographic characteristics of the participants were described in terms of rate and percentage by different functional capability groups.

Second, the binary logistic regression model with an enter method was employed to investigate the relationship between social capital and functional ability. We initially performed a univariate logistic regression model and then performed a logistic regression model adjusted potential covariates (including age, gender, body mass index, residence, living status, marital status, education, smoking status, drinking status, and multimorbidity status). For social capital variables, subjects with a higher level were used as a reference group in all models. For other variables, subjects aged 60–64 years, who were male, having BMI < 18.5 kg/m^2^ (normal weight), residence in urban, living with others, being married/cohabited, reporting high school and above, nonsmoking, nondrinking, and without multimorbidity were grouped as reference. The results of the binary regression logistic analysis were expressed with the odds ratio (OR) and adjusted odds ratio (AOR) and associated 95% confidence interval (95% CI). There was no evidence of multicollinearity in the models according to the variance inflation factor (VIF) result with no factors exceeded the critical value (Table S1 and Table S2).

Third, to further investigate the interactive relationship between social capital and relevant factors associated with functional ability, a classification and regression tree model (CART) was used. This model is a novel method and can be used to examine the complex combinations or interactions among factors and variables that may be ignored in the traditional analytical method [34]. In this analysis, functional ability would be divided into subgroups by the most explanatory independent variables. Any possible interaction and combination with all social capital dimensions and demographic and health-related variables could yield these subgroups. All the variables statistically significant in the univariate regression model were included in the current model. In order to have the optimal classification tree model, an exhaustive CHAID method was selected as the growing method.

All statistical analyses were carried out using SPSS 23.0 statistical software (SPSS Inc., Chicago, IL, USA). A *p*-value < 0.05 was utilized to take statistical significance in this study.

## 3. Results

### 3.1. Results of Descriptive Analysis

The results of the characteristics of participants were shown in Table 1. The prevalence rate of functional disability was about 43% (778 out of 1810). The age of the 1810 participants ranged from 60 to 96 years (71.20 ± 7.51), and most of the subjects were female (57.5%), living in rural area (55.7%), living with others (86.6%), married or cohabited (77.5%), and attended primary school and below (71.3%). About one-third of participants (31.3%) reported having comorbidity. The majority of the subjects had a high social connection (70.7%).

### 3.2. Results of Logistic Analysis

The results of the binary regression logistic analysis of the relationship between social capital and elderly functional ability are presented in Table 2.

In the univariate model, in comparison with reference groups, the odds of being functional disability were increased with age and highest among those with age ≥ 80 years (OR = 5.39; 95% CI: 3.87–7.51), females (OR = 2.10; 95% CI: 1.73–2.55), living in rural area (OR = 2.57; 95% CI: 2.11–3.12), being single (OR = 2.07; 95% CI: 1.65–2.58), with less educational attainment (OR=7.30; 95% CI: 4.90–10.88), with multimorbidity (OR = 2.30; 95% CI: 1.88–2.82). Among six social capital dimensions, in comparison with reference groups, all lower-level social capital was linked to functional disability, except social support that showed no statistical significance.

After adjusting for all covariate variables, in comparison with reference groups, lower social participation (OR = 1.60; 95% CI: 1.26–2.03) and lower social connection (OR = 1.74; 95% CI: 1.34–2.25) were associated with functional disability, which indicated that less social participation and social connection were risk factors for developing functional limitations among older adults. However, social support (OR = 0.73; 95% CI: 0.57–0.94) was inversely associated with functional ability. This adjusted model did not reveal any statistically significant association between trust, cohesion, and reciprocity and functional disability.

### 3.3. Results of Classification and Regression Tree Model

**Figure 1:**
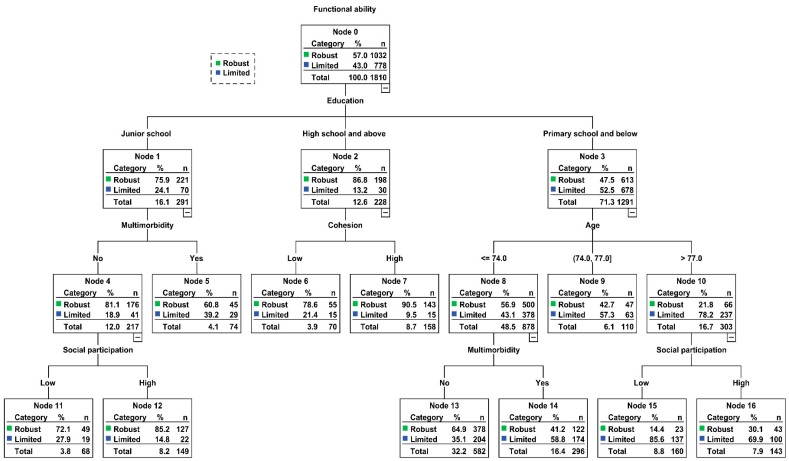
Classification and regression tree model (N = 1810).

The results of CART model are displayed in Figure 1. Functional ability was mainly concerned with education, multimorbidity, cohesion, age, and social participation. Education was the first split factor related to functional function. Moreover, the interactive association among social capital dimensions and various variables were identified.

Among participants with the least educational attainment (primary school and below) (node 3), older age (>77 years) (node 10), and lower social participation (node 15), the possibility of suffering from functional limitation was the highest (node 15).

Those who reported highest educational attainment (high school and above) (node 2) and lower level of cohesion (node 6) had a higher rate of having functional limitations, in comparison with a higher level of cohesion (node 7).

For those with higher educational attainment (junior school) (node 1), without multimorbidity (node 4), and with higher-level social participation (node 12), the proportion of developing functional disability was the least (node 12).

## 4. Discussion

This study examined the association between social capital and elderly functional capability among community-dwelling older adults in Anhui, China. The results demonstrated an association of elderly functional ability with social capital. Specifically, respondents with a higher level of social participation social connection would be heathier in functional ability status. Furthermore, in our study, the interactive relationship between social capital and various variables related to functional ability was firstly determined.

In this study, we found the prevalence rate of functional disability to be 43%, which is consistent with the reported prevalence rate of 43.4% among elderly people aged 60 years and above in Guangxi province, China [9], but higher than the prevalence rate (18%) in Irish elderly people aged 65 and above [35]. This inconsistency may be due to different cultural and historical contexts between two countries as well as different samples and investigation scales. Additionally, our results suggest that the application of a composite scale including both ADL and IADL items to measure individual functional capability status is feasible and reliable [36].

We found variations in findings regarding the relationship between social capital and functional capability status. In line with our findings that elderly people with more social participation had better functional capability status, results from Japan and Indonesia also found that elderly individuals with less social participation had a decline in ADL [4,24,37]. A cohort study from Sweden also found that, in later life, older people who more engaged in more valued social activities were more socially active and physically active, therefore more reported less functional disability [7]. Our study showed that a higher level of social connection was associated with better elderly functional ability, as supported by previous studies reporting that more social connection linked to less likely to have functional limitations [38,39].

According to our analyses, social support was inversely related to functional ability, which suggested that older people with more social support were more likely to be functionally limited. However, findings from Mexico revealed that among community-dwelling older people, those who had fewer social support were more likely to have limitation in at least ADL [40]. A possible reason could be that there were differences in definition and measurement of social support between the two studies. For example, in our study, social support was defined as resources that can be available to the elderly when they were in a difficult time. Four items concerning the frequency of getting support (both subjective and objective) from someone else, organizations or groups were asked to assess the level of social support when they were in need. Nevertheless, in the Mexico study [40], social support was known as connections and contacts that people can obtain emotional, informational, and instrumental support from it. Three types of questions (familial social support networks, extra-familial support networks, and institutional support networks) were utilized to evaluated social support. This variation further highlighted the necessity to determine a universal and consent definition and measurement of social capital [41]. Surprisingly, different from our findings, a study in Shanghai, China found that social support, particularly the family social support did not play a role in the improvement of elderly functional ability [42]. A possible explanation could be that urbanization and rapid economic growth decreased the family resources that provided support for elderly individuals, and community older dwellers prefer to gain support from participating in formal/informal groups, voluntary activities and services in the community/village [43].

Our results indicated that in the logistic regression model, trust, cohesion, and reciprocity were not statistically associated with elderly functional ability, which showed a difference from previous studies. A Brazil study found that the cohesion of elderly participants was associated with their functional disability, while the significant association was not observed for other elements of social capital such as [22]. Results from Japan demonstrated that elderly males with lower-level trust had a higher risk of being decline in ADL [4]. Besides, a cross-sectional study from India found that elderly individuals who having someone to trust were associated with keeping good ADL [44]. Also, findings from the Japan Gerontological Evaluation Study (JAGES) suggested that community social capital (including social cohesion and reciprocity) played a greater role in functional ability improvements [45]. In the present study, the interactive relationship between social capital and various variables related to functional ability was firstly observed.

Most of the previous studies found that lower education [9,11], increased age [10,11], and multimorbidity [3,16,17] were related to poor functional ability. Meanwhile, the role of social capital in elderly health have been noted from multiple studies [22,46]. Although such studies analyzing factors individually related to functional ability are helpful, more comprehensive approaches are needed to examine the extent to which these factors co-combinate and interact to be linked to functional ability. In this study, we utilized CART model to explore the interactive relationship between social capital dimensions and various variables, we found that older adults whose educational attainment was at primary school and below, aged >77 years, and had lower social participation, had the highest risk for developing functional disability. Meanwhile, among older people with higher educational attainment (junior school), without multimorbidity, and with higher-level social participation, the proportion of developing functional disability was the lowest. Interestingly, the role of cohesion was observed in the CART model, which indicated that older people who reported highest educational attainment (high school and above) and lower level of cohesion had a higher rate of having functional limitation when compared with a higher level of cohesion. However, this significance was not observed in the logistic regression analyses. This may imply that the effect of cohesion on elderly functional ability was conditional on the occurrence of other variables, which further indicated the benefit of the CART model. It is the first time that interactive relationship among social capital and other variables was confirmed. These results may aid in appreciating which subsets of older adults are most likely to develop functional disability and shedding light on devising specific and comprehensive programs to prevent functional decline or limitation among community-dwelling older people.

Our study had several strengths. First, we utilized a composite and validated scale including both ADL and IADL items to measure the functional capability status in China, which potentially reduced bias with regard to item complexity and task specificity [47]. Second, the participants of this study were representative with a good response rate. Besides, methodically, we employed the CART model, this is a sophisticated and comprehensive approach, which allows us to explore multiple variables inter-relationships and provide us a straightforward and easily visible tree, which is helpful in the process of decision making. Third, to our knowledge, this is the first study to explore the interactive relationship among social capital and other variables related to functional ability, which provides more insights into designing targeted and effective measures to prevent the onset of elderly functional disability.

However, there are a few limitations that should be acknowledged. First, this was a cross-sectional study, which made it hard to determine causality of social capital and functional ability. Longitudinal study design is warranted for future study. Second, because this study was conducted only in Anhui province, cautions are needed when generalizing our results to other regions or countries. Third, social capital data analyzed in this study were only measured at individual level, we did not include community-level social capital. Future research considering community-level social capital may contribute to a better understanding of the role of social capital.

## 5. Conclusions

Among community-dwelling older adults in Anhui province, some dimensions of social capital were significantly related to elderly functional capability and that the findings do not reveal consistent results across all analytical methods chosen. In particular, a low level of social participation and social connection put the elderly at greater risk of functional disability. Special attention and efforts should be paid to older adults with less educational attainment, with multimorbidity, with advanced age, and lower level of social participation and cohesion. Our findings may be helpful in developing strategies to prevent functional limitations and lessen the associated negative impacts on individuals, families, and society.

## Figures and Tables

**Table 1:** Descriptive results of participants characteristics (N = 1810).

Variables	Total *n* = 1810	Robust *n* = 1032	Limited *n* = 778	χ^2^	*p*-Value *
**Age, years, N (%)**				130.366	<0.001
60–64	399 (22.0)	281 (27.2)	118 (15.2)		
65–69	424 (23.4)	267 (25.9)	157 (20.2)		
70–74	421 (23.3)	262 (25.4)	159 (20.4)		
75–79	282 (15.6)	135 (13.1)	147 (18.9)		
≥80	284 (15.7)	87 (8.4)	197 (25.3)		
**Gender N (%)**				57.518	<0.001
Male	770 (42.5)	518 (50.2)	252 (32.4)		
Female	1040 (57.5)	514 (49.8)	526 (67.6)		
**BMI, kg/m^2^, N (%)**				26.910	<0.001
<18.5	189 (10.4)	88 (8.5)	101 (13.0)		
18.5–22.9	825 (45.6)	439 (42.5)	386 (49.6)		
23.0–27.4	644 (35.6)	411 (39.8)	233 (29.9)		
≥27.5	152 (8.4)	94 (9.1)	58 (7.5)		
**Residence N (%)**				91.924	<0.001
Urban	801 (44.3)	557 (54.0)	244 (31.4)		
Rural	1009 (55.7)	475 (46.0)	534 (68.6)		
**Living status N (%)**				1.418	0.234
Living with others	1567 (86.6)	902 (87.4)	665 (85.5)		
Living alone	243 (13.4)	130 (12.6)	113 (14.5)		
**Marital status N (%)**				41.402	<0.001
Married/Cohabited	1402 (77.5)	856 (82.9)	546 (70.2)		
Single	408 (22.5)	176 (17.1)	232 (29.8)		
**Education N (%)**				173.182	<0.001
Primary school and below	1291 (71.3)	613 (59.4)	678 (87.1)		
Junior school	291 (16.1)	221 (21.4)	70 (9.0)		
High school and above	228 (12.6)	198 (19.2)	30 (3.9)		
**Smoking status N (%)**				43.239	<0.001
Nonsmoking	1412 (78.0)	752 (72.9)	660 (84.8)		
Former smoking	99 (5.5)	59 (5.7)	40 (5.1)		
Current Smoking	299 (16.5)	221 (21.4)	78 (10.0)		
**Drinking status N (%)**				23.469	<0.001
Nondrinking	1484 (82.0)	816 (79.1)	668 (85.9)		
Former drinking	70 (3.9)	35 (3.4)	35 (4.5)		
Current Drinking	256 (14.1)	181 (17.5)	75 (9.6)		
**Multimorbidity N (%)**				66.651	<0.001
No	1244 (68.7)	789 (76.5)	455 (58.5)		
Yes	566 (31.3)	243 (23.5)	323 (41.5)		
**Social participation N (%)**				48.281	<0.001
High	1043 (57.6)	667 (64.6)	376 (48.3)		
Low	767 (42.4)	365 (35.4)	402 (51.7)		
**Social support N (%)**				0.373	0.541
High	906 (50.1)	523 (50.7)	383 (49.2)		
Low	904 (49.9)	509 (49.3)	395 (50.8)		
**Social connection N (%)**				45.604	<0.001
High	1279 (70.7)	794 (76.9)	485 (62.3)		
Low	531 (29.3)	238 (23.1)	293 (37.7)		
**Trust N (%)**				6.069	0.014
High	1023 (56.5)	609 (59.0)	414 (53.2)		
Low	787 (43.5)	423 (41.0)	364 (46.8)		
**Cohesion N (%)**				9.802	0.002
High	1078 (59.6)	647 (62.7)	431 (55.4)		
Low	732 (40.4)	385 (37.3)	347 (44.6)		
**Reciprocity N (%)**				13.368	<0.001
High	978 (54.0)	596 (57.8)	382 (49.1)		
Low	832 (46.0)	436 (42.2)	396 (50.9)		

* Chi-square test.

**Table 2:** Logistic analysis examining associations with functional disability (N = 1810).

	Crude OR (95% CI)	Adjusted OR (95% CI)
**Age, years**		
60–64	(reference)	(reference)
65–69	1.40 (1.05–1.87) *	1.18 (0.85–1.64)
70–74	1.45 (1.08–1.93) *	1.49 (1.06–2.07) *
75–79	2.59 (1.89–3.56) **	2.97 (2.04–4.34) **
≥80	5.39 (3.87–7.51) **	6.01 (4.03–8.97) **
**Gender**		
Male	(reference)	(reference)
Female	2.10 (1.73–2.55) **	1.74 (1.31–2.31) **
**BMI, kg/m^2^**		
18.5–22.9 = normal weight	(reference)	(reference)
<18.5 = underweight	1.31 (0.95–1.79)	0.91 (0.63–1.33)
23.0–27.4 = overweight	0.64 (0.52–0.80) **	0.73 (0.57–0.94) **
≥27.5 = obese	0.70 (0.49–1.00)	0.61 (0.40–0.92) **
**Residence**		
Urban	(reference)	(reference)
Rural	2.57 (2.11–3.12) **	2.29 (1.80–2.91) **
**Living status**		
Living with others	(reference)	(reference)
Living alone	1.18 (0.90–1.55)	0.44 (0.30–0.65) **
**Marital status**		
Married/Cohabited	(reference)	(reference)
Single	2.07 (1.65–2.58) **	1.55 (1.12–2.14) *
**Education**		
High school and above	(reference)	(reference)
Junior school	2.09 (1.31–3.34) *	1.87 (1.12–3.11) *
Primary school and below	7.30 (4.90–10.88) **	4.67 (2.98–7.31) **
**Smoking status**		
Nonsmoking	(reference)	(reference)
Former smoking	0.77 (0.51–1.17)	0.93 (0.52–1.64)
Current Smoking	0.40 (0.30–0.53) **	0.52 (0.36–0.76) *
**Drinking status**		
Nondrinking	(reference)	(reference)
Former drinking	1.22 (0.76–1.97)	2.03 (1.05–3.91) *
Current Drinking	0.51 (0.38–0.68) **	0.90 (0.62–1.29)
**Multimorbidity**		
No	(reference)	(reference)
Yes	2.30 (1.88–2.82) **	2.32 (1.83–2.95) **
**Social participation**		
High	(reference)	(reference)
Low	1.95 (1.62–2.36) **	1.60 (1.26–2.03) **
**Social support**		
High	(reference)	(reference)
Low	1.06 (0.88–1.28)	0.73 (0.57–0.94) *
**Social connection**		
High	(reference)	(reference)
Low	2.02 (1.64–2.47) **	1.74 (1.34–2.25) **
**Trust**		
High	(reference)	(reference)
Low	1.27 (1.05–1.53) *	0.95 (0.73–1.24)
**Cohesion**		
High	(reference)	(reference)
Low	1.35 (1.12–1.64) *	0.90 (0.69–1.18)
**Reciprocity**		
High	(reference)	(reference)
Low	1.42 (1.18–1.71) **	1.16 (0.89–1.52)

Note: Adjusted age, gender, BMI, residence, living status, marital status, education, smoking status, drinking status, and multimorbidity status. * *p* < 0.05. ** *p* < 0.001.

## Supplementary Materials

The following are available online at https://www.mdpi.com/1660-4601/17/8/2775/s1, Table S1: Results of multicollinearity examination, Table S2: Correlations results between the variables.
